# Outcomes of the Valve-Sparing Root Replacement Procedure with Partial Upper Sternotomy

**DOI:** 10.3390/jcdd8110154

**Published:** 2021-11-09

**Authors:** Bin Hou, Rui Zhao, De Wang, Wei Wang, Zhenhua Zhao, Xiaogang Sun, Xiangyang Qian, Cuntao Yu

**Affiliations:** Department of Cardiovascular Surgery, Fuwai Hospital, National Centre for Cardiovascular Diseases, Peking Union Medical College and Chinese Academy of Medical Sciences, Beijing 100037, China; hbfw2009@163.com (B.H.); zhaorui@fuwai.com (R.Z.); wangdecn@163.com (D.W.); 13426095145@139.com (W.W.); 13488738631@139.com (Z.Z.); xiaogangsun2006@vip.sina.com (X.S.); m13701097213@163.com (X.Q.)

**Keywords:** minimally invasive, valve-sparing root replacement, DAVID procedure, aortic aneurysm, propensity-matched analysis

## Abstract

Due to better postoperative convalescence and quality of life, experienced centers focus on minimally invasive surgical techniques and approaches, but this approach is not routinely performed for valve-sparing root replacement procedures. The purpose of this study was to assess the safety and feasibility of valve-sparing root replacement via partial upper sternotomy. Between January 2016 and April 2021, 269 patients underwent a valve-sparing root replacement procedure, and partial upper sternotomy was performed in 52 patients. The clinical outcomes of the partial upper sternotomy (PUS) and complete sternotomy (CS) groups, including mortality, degree of aortic insufficiency, blood loss and consumption of blood products, postoperative complications, and hospitalization expenses, were compared. The Kaplan–Meier method was used to assess the degree of aortic regurgitation. Propensity score matching was performed as a sensitivity analysis. There was only one in-hospital death (in the CS group, *p* = 1) and no postoperative moderate to severe aortic insufficiency in either group. The blood loss and consumption of blood products in the PUS group were also lower than in the CS group, especially for plasma use. Regarding the need for re-exploration because of bleeding, acute kidney injury, pericardial pleural effusion, drainage volume within the first 24 h, mechanical ventilation time, and arrhythmia, the two groups were comparable. Patients in the CS group showed a longer ICU time (74.20 ± 47.21 vs. 50.9 30.16 h, *p* = 0.001) and higher hospitalization expenses (135,649.52 ± 29,992.21 vs. 123,380.15 ± 27,062.82 yuan, *p* < 0.001). None of the patients died or reoperated during the follow-up. Freedom from moderate or severe aortic insufficiency remained comparable after matching (*p* = 0.97). Minimally invasive valve-sparing aortic replacement via partial upper sternotomy can be safely performed in selected patients.

## 1. Introduction

The valve-sparing root replacement procedure (VSRR) is a well-known technique in selected patients with aortic root pathology and is associated with improved quality of life compared with the Bentall procedure [1,2,3]. Due to better postoperative convalescence and quality of life, experienced centers focus on minimally invasive surgical techniques and approaches. Partial upper sternotomy (PUS) is an established approach for aortic valve replacement and aortic surgery with excellent clinical outcomes [4,5]. In our institute, we have utilized partial upper sternotomy to perform VSRR since 2016 in select cases and achieved good early outcomes. In this study, we present our clinical experience with VSRR procedures with partial upper sternotomy and compare them with complete sternotomy (CS).

## 2. Materials and Methods

### 2.1. Study Population and Follow Up

**Figure 1:**
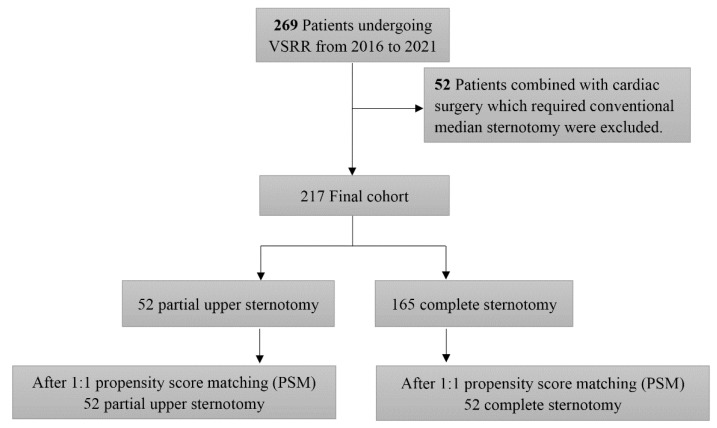
Study design: summary of the inclusion and exclusion criteria of the study population.

This was a retrospective study and was approved by the Institutional Review Board at Fuwai Hospital. In total, 269 patients underwent the VSRR procedure alone or in combination with other procedures for aortic aneurysm or aortic dissection between January 2016 and April 2021 at Fuwai Hospital. At the beginning of the series, CS was performed, and we moved on to PUS for the VSRR procedure after gaining more experience. Patients with coronary artery disease, mitral valve, and tricuspid valve disease who required conventional median sternotomy were excluded (*n* = 52). Partial upper sternotomy was performed in 52 patients (Figure 1). The decision to use VSRR was made if the preoperative transesophageal echocardiographic (TEE) scans showed no calcified aortic valve cusps, and the presence of intact and tender cusps was verified intraoperatively. PUS was implemented at the surgeon’s discretion. After discharge, the patient’s condition was checked through telephone calls if they did not attend the scheduled clinic visit.

### 2.2. Surgical Technique

**Figure 2:**
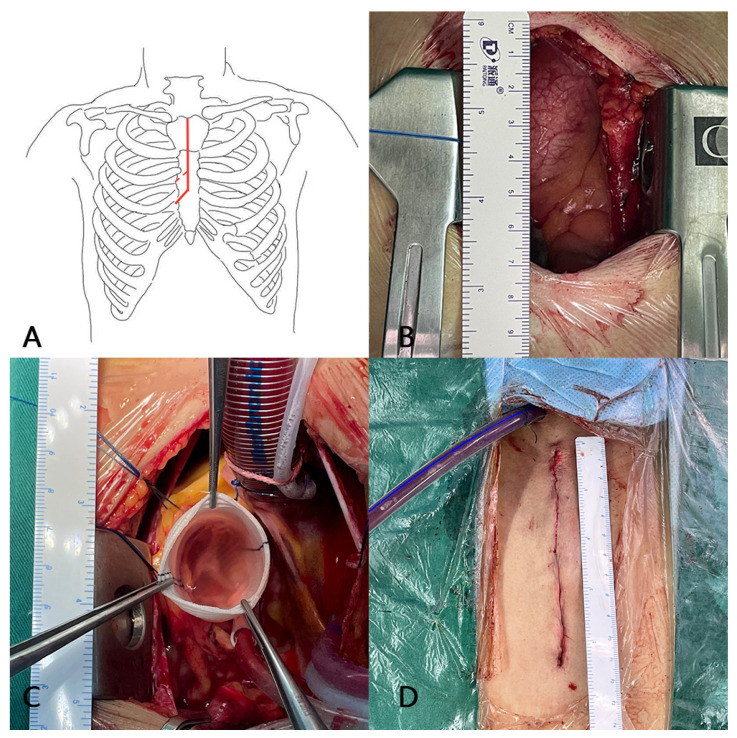
Minimally invasive access through partial upper sternotomy for the VSRR procedure showing the pattern (**A**), incision (**B**), aortic valve assessed by a water injection experiment (**C**), and suture (**D**).

The skin incision was performed longitudinally 8 to 12 cm from one finger below the sternal notch to the third intercostal space level. Afterward, the sternum was divided in a J-shaped manner from the sternal notch down to the right fourth intercostal space (Figure 2). The right internal mammary artery was given specific attention to protect it from injury. We initiated mild hypothermic cardiopulmonary bypass with a target nasal temperature of approximately 32 to 33 °C. Alternatives for arterial cannulation were the ascending aorta or the femoral artery depending on the diameter of the distal ascending aorta. Venous cannulation was performed through the right atrium. Left heart drainage was placed through the right upper pulmonary vein. Cold cardioplegic solution (Thomas solution) was administered in an antegrade fashion directly into the coronary ostia through a tube every 30 min.

In our institute, we perform the reimplantation technique and choose straight tubular grafts without Valsalva sinuses for most patients. The size of the graft is based on the patient’s body weight and the diameter of the aortic valve annulus. The number of sutures below the aortic valve annulus is 6 or 7, usually sewing with Gore-Tex CV5. The sutures are tied to shape the bottom portion of the aorta graft with slight contraction of the annulus. Anastomosis of the residual sinus wall and artificial graft as well as coronary artery buttons reattached to the aorta graft are sewn continuously with 5-0 Prolene sutures. Three commissures were secured to the graft with pledget-supported 5-0 double-armed polypropylene sutures. Next, starting at the bottom of one sinus, the remnant of the aortic sinus wall was sutured to the graft with a continuous 5-0 double-armed polypropylene suture from bottom of the sinus to the commissure. Each stitch should carry both graft vessel and aortic sinus wall. When suturing arrived at the commissure, a knot was tied with one of the pledget-supported 5-0 double-armed polypropylene described above. Distal aorta graft anastomosis to the ascending aorta is finished with 4-0 or 5-0 Prolene sutures. In some patients with aortic valve regurgitation, cusp plication is required. The effect of aortic valvuloplasty during aortic cross clamp is assessed by a water injection experiment and re-evaluated by TEE after cardiac resuscitation. More than moderate valvular regurgitation is unacceptable and requires repair again. For patients with extensive aortic arch procedures, as the nasopharyngeal temperature is lowered to 25 °C, hypothermic circulatory arrest is performed, and antegrade cerebral perfusion (ACP) is instituted at a flow rate of approximately 5 to 10 mL/kg/min.

### 2.3. Statistical Analysis

Statistical analyses were performed with R 4.0.5. A 2-tailed *p* value < 0.05 indicated statistical significance. Continuous variables were tested for normal distribution using the Shapiro–Wilk normality test. Continuous variables with a normal distribution are expressed as the mean ± standard deviation and were compared using the t test. Nonnormally distributed continuous data are summarized as the median (interquartile range, IQR) and were compared using the Mann–Whitney test. Categorical variables are expressed as counts and composition ratios and were compared using the chi-square test or Fisher’s exact test as appropriate. To compensate for differences in preoperative patient characteristics, a 1:1 propensity match was performed with the R package MatchIt using the following parameters: age, male sex, hypertension, diabetes, type A dissection, Marfan syndrome, preoperative grade of aortic insufficiency (AI), and valve-sparing root replacement only. Moderate to severe aortic insufficiency was calculated according to the Kaplan–Meier method.

## 3. Results

### 3.1. Patient Baseline Characteristics

**Figure 3:**
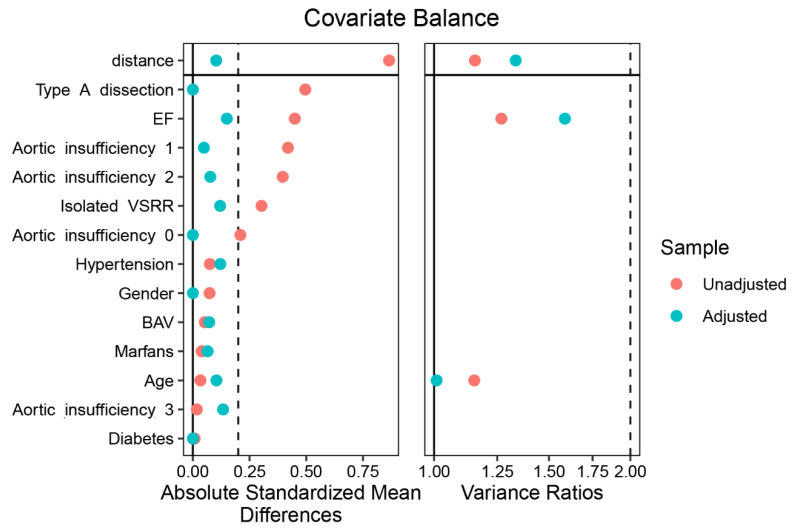
Standardized mean differences for sternotomy pairwise comparisons via the LOVE plot.

Baseline characteristics according to different types of sternotomy are shown in Table 1. There was no significant difference in age between the PUS group and the CS group (45.48 ± 12.97 vs. 45.89 ± 12.09 years, *p* = 0.842). Approximately 84.6% of the minimally invasive operated patients were males (44/52) vs. 87.3% of the patients in the complete sternotomy group (144/165) (*p* = 0.797). Concerning the presence of Marfan syndrome, bicuspid aortic valve, hypertension, and diabetes, the two groups did not differ. Regarding echocardiographic findings, patients in the PUS group more often had moderate or severe aortic insufficiency, and this difference reached significance (moderate, 51.9% vs. 32.1%; severe, 25% vs. 24.2%, *p* = 0.036). After matching, the differences in age, male sex, hypertension, diabetes, type A dissection, Marfan syndrome, and preoperative grade of aortic insufficiency did not remain significant (Figure 3).

### 3.2. Perioperative Data

Patients in the complete sternotomy group more frequently received an additional total arch procedure (15.2% vs. 1.9%, *p* = 0.021) and frozen elephant trunk (12.7% vs. 1.9%, *p* = 0.047). Operation time (5.13 ± 1.06 vs. 6.74 ± 1.73 h, *p* < 0.001), cardiopulmonary bypass (CPB) time (181.84 ± 55.96 vs. 138.98 ± 30.94 h, *p* < 0.001), and aortic cross clamping (ACC) time (145.88 ± 38.54 vs. 110.56 ± 19.91 h, *p* < 0.001) were significantly shorter in the PUS group, and even after matching, differences were still present (Table 2). In addition, the blood loss and consumption of blood products in the PUS group were less than those in the CS group, especially for plasma use (0 [0,0] vs. 0 [0,400] mL, *p* < 0.001, median [IQR]). Even after matching, there was still a significant difference.

### 3.3. Postoperative Mortality, Morbidity, and Hospitalization Expenses

There was only one in-hospital death (in the CS group, *p* = 1) and no postoperative moderate to severe aortic insufficiency in either group (Table 3). Regarding the need for re-exploration because of bleeding, acute kidney injury (KDIGO criteria), pericardial pleural effusion, drainage volume within the first 24 h, mechanical ventilation time, and arrhythmia, the two groups were comparable (*p* = 1; *p* = 0.766; *p* = 1; *p* = 0.211; *p* = 0.221; *p* = 0.588). Patients in the CS group showed a longer ICU time (74.20 ± 47.21 vs. 50.94 ± 30.16, *p* = 0.001) and higher hospitalization expenses (135,649.52 ± 29,992.21 vs. 123,380.15 ± 27,062.82 yuan, *p* < 0.001)

### 3.4. Follow-Up and Characteristics of Echocardiography

**Figure 4:**
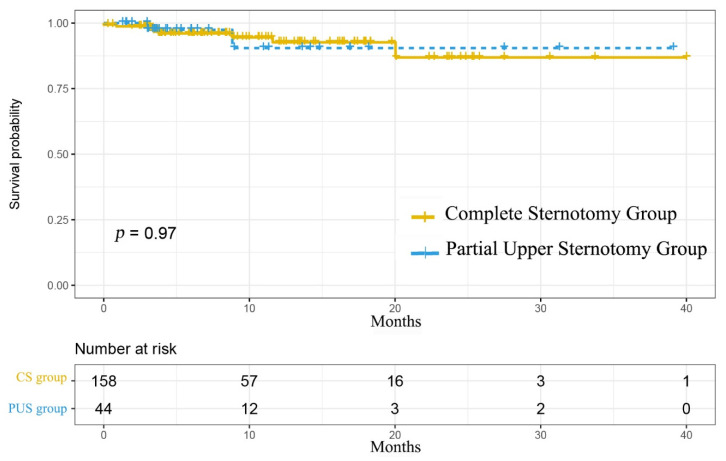
Kaplan–Meier curve for freedom from reoperation or moderate to severe aortic insufficiency.

The cumulative follow-up was 9 ± 7.75 months (range: 1–40 months), with 91% completeness. None of the patients died or were reoperated during the follow-up. Nine patients developed moderate or severe aortic insufficiency during follow-up. Two of them were in the PUS group (4.5%), and seven were in the CS group (4.4%). Freedom from moderate or severe aortic insufficiency remained comparable after matching (*p* = 0.97) (Figure 4). In addition, we compared the preoperative, postoperative, and latest follow-up echocardiographic data between the two groups and found that there was no significant difference (Table 4).

## 4. Discussion

Our study showed that compared with complete sternotomy, partial upper sternotomy has the same curative effect with the VSRR procedure for select patients, and it can reduce the use of blood products and shorten surgery time, cardiopulmonary bypass time, aortic cross clamping time, and stay time in the ICU. The same effect was consistent in propensity-matched cohort analysis.

Several studies have demonstrated excellent long-term results with VSRR procedures, which are a preferable option for patients presenting with aortic root disease with or without aortic regurgitation [2,6,7]. In the late 1990s, different minimally invasive approaches were introduced for aortic valve surgery to reduce surgical trauma and to improve the postoperative course [8]. Most of the studies of valve-sparing root replacement procedures were reported as complete sternotomies, while minimally invasive procedures have not been carried out on a large scale. This may be due to the high technical requirements of the VSRR procedure for the surgeon, which makes most doctors choose conventional complete sternotomy to ensure good exposure. Until 2015, Shrestha et al. [9]. reported a limited number of pilot projects in which the ascending aorta and the aortic root were exposed via an upper J mini-sternotomy (up to the third intercostal space) after gaining experience with more than 500 valve-sparing root replacement procedures via full sternotomy, as well as more than 200 minimally invasive aortic valve replacements (AVRs). With a 30-day mortality of 0% and no severe AI after a 1-year follow-up, we can confirm the good results for the minimally invasive VSRR procedure. After that, many researchers tried to perform the VSRR procedure via minimally invasive sternotomy, but the sample sizes were limited [10,11,12]. Another meta-analysis of 1101 cases of minimally invasive aortic surgery and 1405 cases of median complete sternotomy showed that minimally invasive aortic surgery may be related to the improvement of early clinical outcomes [13]. Therefore, the VSRR procedure via partial upper sternotomy is a feasible method, and our study also achieved the same results as previous studies. In addition, we also conducted a cost-benefit analysis and found that partial upper sternotomy can reduce the cost of patients.

The reduction in the surgery time, CPB time, and ACC time for PUS group patients in our study is consistent with current trends in minimally invasive valve-sparing root replacement procedures [12]. Since 2016, our center has tried to perform all kinds of aortic surgery via partial upper sternotomy, including aortic valve replacement, ascending aorta replacement, the Bentall procedure, and the VSRR procedure. All minimally invasive cases were performed by experienced surgeons who had completed enough cases of partial upper sternotomy with other aortic surgeries. In addition, due to the reduction of intraoperative blood loss, the difficulty associated with hemostasis during the process of sternal closure is also reduced. Minimally invasive incision also reduces the length of the incision and the use of the number of wires, which to some extent speeds up the process of surgery. Shrestha et al. [9] found that only after a notable amount of experience (>100 operations) should the surgeon move on to the next step of minimal access ascending aortic replacements with or without AVR, and only after gaining enough experience should the surgeon move to minimal access valve-sparing aortic root replacement. We also started with AVR, followed by the Bentall procedure, ascending aortic replacement, wheat procedure, and finally the VSRR procedure. We initially performed partial upper sternotomy up to the fourth intercostal space and then changed to the third intercostal space after gaining enough experience. In our cohort, two patients were enrolled before 2018 who were younger with a moderate degree of aortic sinus dilatation and aortic regurgitation, and the remaining 50 patients were enrolled continuously without special selection after this. We found that when choosing select patients at the beginning of the learning curve, minimally invasive surgery can obtain early results that are not inferior to conventional median sternotomy, with less bleeding, less pain, and faster postoperative recovery.

We also found that valve-sparing root replacement via partial upper sternotomy was associated with a reduction in the length of ICU stay. This finding is consistent with the current literature for minimal access cardiac surgery [13,14,15,16]. Prolonged ICU stay is associated with postoperative mortality and complications [17,18], so minimizing this would be a huge advantage of partial upper sternotomy. This could be a consequence of attenuated postoperative pain, although the lack of data on this outcome does not allow us to make firm conclusions [12]. Shortening the ICU stay can also reduce the cost of patients [19], which is very important in our current medical environment, so partial upper sternotomy can reduce physical pain and economic pressure.

As this study is a retrospective nonrandomized trial, the groups are not comparable in several aspects, especially for the factors about surgeons, but this is an unavoidable factor in many similar studies. Many cases in the PUS group were operated after the surgeons had performed enough CS surgery and surgeons may have chose some patients with relatively mild conditions for the PUS procedure, so we used the propensity score to reduce the influence from patients. Although there are no unacceptable results in our study, in fact, a minimally invasive approach can be either very successful or lead to real disaster sequences even in selected patients. Therefore, we should first carry out this operation on the premise of ensuring the safety of patients. In addition, our sample is limited, but this is also the largest group of VSRR procedures performed via different sternotomies. Finally, the follow-up period is limited. During the follow-up period, aortic regurgitation in some patients progressed, but the indication for intervention had not yet been reached. Further studies with larger sample sizes and longer follow-up times will be carried out.

## 5. Conclusions

The valve-sparing root replacement procedure with partial upper sternotomy for select patients with aortic root aneurysm and aortic dissection is a feasible procedure with minor valve-related morbidity and mortality at the mid-term follow-up. The intraoperative application of blood products and ICU stay were significantly lower and shorter, respectively, in the partial upper sternotomy group. In addition, partial upper sternotomy can reduce the cost of patients. However, a comparison of long-term follow-up data in both groups is necessary.

## Figures and Tables

**Table 1:** Preoperative characteristics of the total cohort and propensity-matched cohorts.

	Total Cohort (*n* = 217)					Propensity-Matched Cohort (*n* = 104)			
	Overall	Complete Sternotomy	Partial Upper Sternotomy	*p*	SMD	Complete Sternotomy	Partial Upper Sternotomy	*p*	SMD
	*n* = 217	*n* = 165	*n* = 52			*n* = 52	*n* = 52		
Age, years (mean (SD))	45.58 (12.74)	45.48 (12.97)	45.89 (12.09)	0.842	0.032	44.63 (12.03)	45.89 (12.09)	0.595	0.104
Gender, *n* (%)	188 (86.6)	144 (87.3)	44 (84.6)	0.797	0.077	44 (84.6)	44 (84.6)	1	<0.001
BMI (mean (SD))	24.68 (3.54)	24.83 (3.57)	24.22 (3.41)	0.282	0.174	25.34 (3.58)	24.22 (3.41)	0.107	0.319
Type A dissection, *n* (%)	27 (12.4)	27 (16.4)	0 (0.0)	0.004	0.626	0 (0)	0 (0)	1	<0.001
Marfan syndrome, *n* (%)	19 (8.8)	14 (8.5)	5 (9.6)	1	0.039	6 (11.5)	5 (9.6)	1	0.063
BAV, *n* (%)	19 (8.8)	15 (9.1)	4 (7.7)	0.976	0.05	5 (9.6)	4 (7.7)	1	0.068
Hypertension, *n* (%)	81 (37.3)	63 (38.2)	18 (34.6)	0.765	0.074	21 (40.4)	18 (34.6)	0.685	0.119
Diabetes, *n* (%)	4 (1.8)	3 (1.8)	1 (1.9)	1	0.008	1 (1.9)	1 (1.9)	1	<0.001
Aortic insufficiency, *n* (%)				0.036	0.483			0.917	0.14
None	15 (6.9)	13 (7.9)	2 (3.8)			2 (3.8)	2 (3.8)		
Mild	69 (31.8)	59 (35.8)	10 (19.2)			11 (21.2)	10 (19.2)		
Moderate	80 (36.9)	5 (32.1)	27 (51.9)			29 (55.8)	27 (51.9)		
Severe	53 (24.4)	40 (24.2)	13 (25.0)			10 (19.2)	13 (25.0)		

SMD, standardized mean difference; BMI, body mass index; BAV, bicuspid aortic valve.

**Table 2:** Perioperative data of the total cohort and propensity-matched cohort.

	Total Cohort (*n* = 217)				Propensity-Matched Cohort (*n* = 104)			
	Complete Sternotomy	Partial Upper Sternotomy	*p*	SMD	Complete Sternotomy	Partial Upper Sternotomy	*p*	SMD
	*n* = 165	*n* = 52			*n* = 52	*n* = 52		
Isolated VSRR, *n* (%)	130 (78.8)	46 (88.5)	0.177	0.264	48 (92.3)	46 (88.5)	0.739	0.131
Partial arch replacement, *n* (%)	10 (6.1)	4 (7.7)	0.925	0.065	3 (5.8)	4 (7.7)	1	0.077
Total arch repair, *n* (%)	25 (15.2)	1 (1.9)	0.021	0.487	1 (1.9)	1 (1.9)	1	<0.001
Frozen elephant trunk, *n* (%)	21 (12.7)	1 (1.9)	0.047	0.424	0 (0.0)	1 (1.9)	1	0.198
Surgery time, h (mean (SD))	6.74 (1.73)	5.13 (1.06)	<0.001	1.123	6.40 (1.42)	5.13 (1.06)	<0.001	1.015
Blood loss, mL (median [IQR])	800 [780,800]	710 [660,800]	0.025	0.275	800 [761.25,800]	710 [660,800]	0.275	0.129
CPB time, min (mean (SD))	181.84 (55.96)	138.98 (30.94)	<0.001	0.948	170.71 (44.65)	138.98 (30.94)	<0.001	0.826
ACC time, min (mean (SD))	145.88 (38.54)	110.56 (19.91)	<0.001	1.151	137.81 (29.20)	110.56 (19.91)	<0.001	1.09
Graft diameter, mm (mean (SD))	28.73 (1.62)	28.50 (1.36)	0.36	0.153	28.73 (1.54)	28.50 (1.36)	0.42	0.159
RBC input (median [IQR])	0 [0.2]	0 [0,0]	0.018	0.387	0 [0.2]	0 [0,0]	0.114	0.276
Plasma input, ml (median [IQR])	0 [0,400]	0 [0,0]	0.001	0.444	0 [0,400]	0 [0,0]	0.023	0.34
PLT input (median [IQR])	1 [1,1]	1 [0,1]	0.069	0.32	1 [1,1]	1 [0,1]	0.462	0.119

VSSR, Valve-Sparing Root Replacement; CPB, cardiopulmonary bypass; ACC, aortic cross clamping; RBC, red blood cell; PLT, platelet.

**Table 3:** Postoperative mortality, morbidity, and hospitalization expenses of the total cohort and propensity-matched cohort.

	Total Cohort (*n* = 217)				Propensity-Matched Cohort (*n*= 104)			
	Complete Sternotomy	Partial Upper Sternotomy	*p*	SMD	Complete Sternotomy	Partial Upper Sternotomy	*p*	SMD
	*n* = 165	*n* = 52			*n* = 52	*n* = 52		
In hospital mortality, *n* (%)	1 (0.6)	0 (0.0)	1	0.11	0 (0.0)	0 (0.0)	1	<0.001
Hospitalization expenses	152,075.56 (58,064.36)	123,380.15 (27,062.82)	0.001	0.633	135,649.52 (29,992.21)	123,380.15 (27,062.82)	0.031	0.43
ICU time, h (mean (SD))	74.20 (47.21)	50.94 (30.16)	0.001	0.587	70.25 (48.65)	50.94 (30.16)	0.017	0.477
Mechanical ventilation, h (mean (SD))	18.98 (25.81)	14.50 (8.33)	0.221	0.233	18.26 (36.23)	14.50 (8.33)	0.468	0.143
Drainage Volume 24 h, ml (mean (SD))	404.42 (225.52)	361.54 (177.22)	0.211	0.211	375.19 (216.35)	361.54 (177.22)	0.726	0.069
Postoperative moderate to severe AI, *n* (%)	0 (0.0)	0 (0.0)	1	<0.001	0 (0.0)	0 (0.0)	1	<0.001
Re exploration, *n* (%)	1 (0.6)	0 (0.0)	1	0.11	0 (0.0)	0 (0.0)	1	<0.001
Acute kidney injury, *n* (%)	3 (1.8)	0 (0.0)	0.766	0.192	0 (0.0)	0 (0.0)	1	<0.001
Arrhythmia, *n* (%)	4 (2.4)	0 (0.0)	0.588	0.223	2 (3.8)	0 (0.0)	0.475	0.283
Pericardial pleural effusion, *n* (%)	6 (3.6)	2 (3.8)	1	0.011	4 (7.7)	2 (3.8)	0.674	0.166

SMD, standardized mean difference; ICU, intensive care unit; AI, aortic insufficiency.

**Table 4:** Echocardiographic data of the propensity-matched cohort.

	Overall	Complete Sternotomy	Partial Upper Sternotomy	*p*
**Preoperative**	*n* = 104	*n* = 52	*n* = 52	
Annulus diameter, mm (mean (SD))	26.65 (2.89)	26.67 (3.07)	26.63 (2.73)	0.937
Sinus diameter, mm (mean (SD))	51.92 (6.82)	52.58 (7.27)	51.27 (6.33)	0.33
Ascending aorta diameter, mm (mean (SD))	42.51 (8.36)	42.87 (8.88)	42.15 (7.87)	0.666
EF, % (mean (SD))	63.39 (3.82)	63.08 (3.37)	63.71 (4.24)	0.4
LVEDD, mm (mean (SD))	56.51 (9.83)	56.37 (11.57)	56.65 (7.84)	0.882
Moderate to severe AI, *n* (%)	79 (76.0)	39 (75.0)	40 (76.9)	1
**Postoperative (before Discharge from Hospital)**	*n* = 104	*n* = 52	*n* = 52	
Aortic Annulus diameter, mm (mean (SD))	21.68 (4.57)	22.82 (2.45)	20.74 (5.63)	0.073
Sinus diameter, mm (mean (SD))	28.56 (6.00)	30.11 (3.54)	27.39 (7.16)	0.075
Ascending aorta diameter, mm (mean (SD))	29.15 (2.96)	28.71 (3.22)	29.56 (2.66)	0.154
EF, % (mean (SD))	60.62 (4.44)	60.56 (4.67)	60.67 (4.24)	0.895
LVEDD, mm (mean (SD))	48.75 (6.37)	48.83 (5.53)	48.67 (7.16)	0.903
Moderate to severe AI, *n* (%)	0 (0)	0 (0)	0 (0)	1
**Latest Follow Up**	*n* = 81	*n* = 44	*n* = 37	
Annulus diameter, mm (mean (SD))	22.62 (2.42)	22.71 (2.79)	22.50 (1.85)	0.724
Sinus diameter, mm (mean (SD))	29.58 (3.09)	29.56 (3.32)	29.61 (2.81)	0.944
Ascending aorta diameter, mm (mean (SD))	29.05 (2.54)	28.91 (2.89)	29.23 (2.06)	0.582
EF, % (mean (SD))	63.01 (5.32)	63.77 (5.47)	62.11 (5.07)	0.162
LVEDD, mm (mean (SD))	49.84 (7.61)	48.64 (9.65)	51.27 (3.66)	0.121
Moderate to severe AI, *n* (%)	5 (6.2)	3 (6.8)	2 (5.4)	1

EF, ejection fraction; LVEDD, left ventricular end-diastolic diameter; AI, aortic insufficiency.

## Data Availability

Data will not be made available.
